# Identification of *Alternaria alternata* Mycotoxins by LC-SPE-NMR and Their Cytotoxic Effects to Soybean (*Glycine max*) Cell Suspension Culture

**DOI:** 10.3390/molecules18032528

**Published:** 2013-02-26

**Authors:** Gezimar D. de Souza, Axel Mithöfer, Cristina Daolio, Bernd Schneider, Edson Rodrigues-Filho

**Affiliations:** 1 Laboratório de Bioquímica Micromolecular de Micro-organismos, Departamento de Química, Universidade Federal de São Carlos—UFSCar, Rodovia Washington Luiz, Km 235, 676 São Carlos-SP, Brazil; E-Mail: edson@dq.ufscar.br; 2 Department of Bioorganic Chemistry, Max Planck Institute for Chemical Ecology; Hans-Knöll-Straße 8, Jena D-07745, Germany; E-Mail: amithoefer@ice.mpg.de; 3 Accert Chemistry and Biotechnology Inc., Rua Alfredo Lopes 1717, São Carlos-SP, Brazil; 4 Biosynthesis/NMR Research Group, Max Planck Institute for Chemical Ecology, Hans-Knöll-Straße 8, Jena D-07745, Germany; E-Mails: cristina.daolio@bruker-biospin.de (C.D.); schneider@ice.mpg.de (B.S.); 5 NMR Applications Group, Bruker Biospin GmbH, Silberstreifen 4, Rheinstetten 76287, Germany

**Keywords:** LC-NMR, LC-SPE-NMR, mass spectrometry, alternariol, *Alternaria*, soybean, *Glycine max*, cell cultures, cytotoxicity

## Abstract

This present work describes the application of liquid chromatograpy-solid phase extraction-nuclear magnetic resonance spectroscopy to analyse *Alternaria alternata* crude extracts. Altenusin (**1**), alternariol (**2**), 3'-hydroxyalternariol monomethyl ether (**3**), and alternariol monomethyl ether (**4**), were separated and identified. High-resolution mass spectrometry confirmed the proposed structures. The cytotoxic effects of these compounds towards plants were determined using soybean (*Glycine max*) cell cultures as a model. EC_50_ values which range from 0.11 (±0.02) to 4.69 (±0.47) μM showed the high cytotoxicity of these compounds.

## 1. Introduction

Metabolites synthesized by plant pathogens are usually associated with disease symptoms [1]. In some cases host-pathogen interactions provide a favorable environment and source for the production of many different compounds, which can be employed in areas of economical relevance. During the last three decades the presence of mycotoxins in citrus plants symptomatic of Alternaria Brown Spot disease has caused several problems for citriculture worldwide. This disease is caused by the phytopathogenic fungus *Alternaria alternata pv. citri* [2]. It promotes lesions on young leaves, branches, and on fruits of tangerines, attacking almost the whole plant. On fruits it boosts lesions that can vary from minute spots to large craters [2,3,4]. Eruptions sometimes are formed and can be dislodged, generating irregular surfaces, reducing yields, and diminishing the marketability of fruits [3].

**Figure 1:**
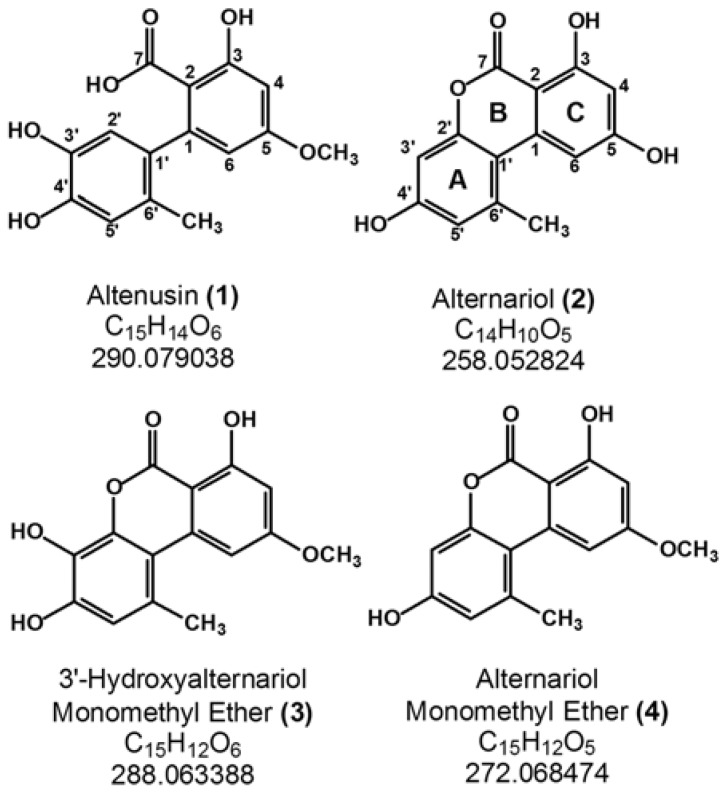
Chemical structures of alternariols **1**–**4**.

Magnani *et al.* [5] employed liquid chromatography-tandem mass spectrometry (LC-MS/MS) for quantification of two *Alternaria* mycotoxins, alternariol (**2**) and alternariol monomethyl ether (**4**) (Figure 1) in different parts of tangerines (*Citrus reticulata*). Recently, Asam *et al.* [6] used isotope dilution assays to detect alternariols in beverages. Other authors developed strategies to extracted simultaneously different types of mycotoxins produced not only by *Alternaria*, but also for other fungi [7,8,9]. These mycotoxins, including those produced by *Alternaria* species, are toxic to both humans and animals [10,11]. Therefore, it is of interest to develop analytical methods for detection and identification of such compounds.

Regarding detection and quantification of mycotoxins in complex matrices, LC-MS/MS is one of the best approaches since it provides high sensitivity and specificity [5]. However, its power for structure elucidation of organic compounds is limited due to the basic principle of such technique. In mass spectrometry the sample molecules are first ionized to obtain fragments of the ions of varied mass [12]. Although fragmentation patterns are somewhat characteristic and predictable for most classes of compounds, *de novo* interpretation without any pre-knowledge is challenging, if not totally impossible, due to the high molecular diversity and many similar compound structures [13]. Even using high-resolution mass spectrometry (HRMS) such task is still hard and time-consuming.

Compared with MS, nuclear magnetic resonance (NMR) spectroscopy yields relatively low-sensitivity measurements, with limits of detection on the order of 10 μM or a few nmol at high fields using new cryoprobes [14]. Nevertheless, NMR is usually preferred for structure elucidation of natural compounds. As NMR spectroscopy is one of the most powerful analytical methods for identification and structure elucidation of organic compounds [15], it is interesting to use the hyphenation between HPLC and NMR in studies involving complex matrices.

Although this coupling is known for more than two decades, improvements in solvent suppression pulse sequences and sensitivity [16] have boosted its application in natural product chemistry. Hyphenation between HPLC, post column solid-phase extraction, and NMR (LC-SPE-NMR) has emerged as a very promising technique for structure elucidation [17,18]. Compounds separated by HPLC are transferred one by one to SPE cartridges in order to remove the non-deuterated mobile phase from the analyte. Subsequently, cartridges are dried by a nitrogen stream, analytes are desorbed with a small volume of deuterated solvent, and transferred through a capillary into the NMR flow cell [19]. The whole process can be carried out under full automation.

This procedure requires minimal quantity of sample and the risk of analyte degradation is minimized. The fraction that is analyzed by NMR can be also recovered for further spectroscopic analysis or bioassays. Moreover, the reproducible NMR measurement conditions allow direct comparison with spectra from conventional off-line NMR equipment [20].

This investigation describes the application of LC-SPE-NMR to identify mycotoxins produced by a pathogenic *Alternaria alternata* strain, which was isolated from tangerines exhibiting Alternaria Brown Spot symptoms. This appears to be the first report concerning the use of a NMR-based coupling method to identify *Alternaria* mycotoxins in complex matrices as well as to report cytotoxic effects of alternariols to plant cells, in particular soybean cell suspension culture.

## 2. Results and Discussion

**Figure 2:**
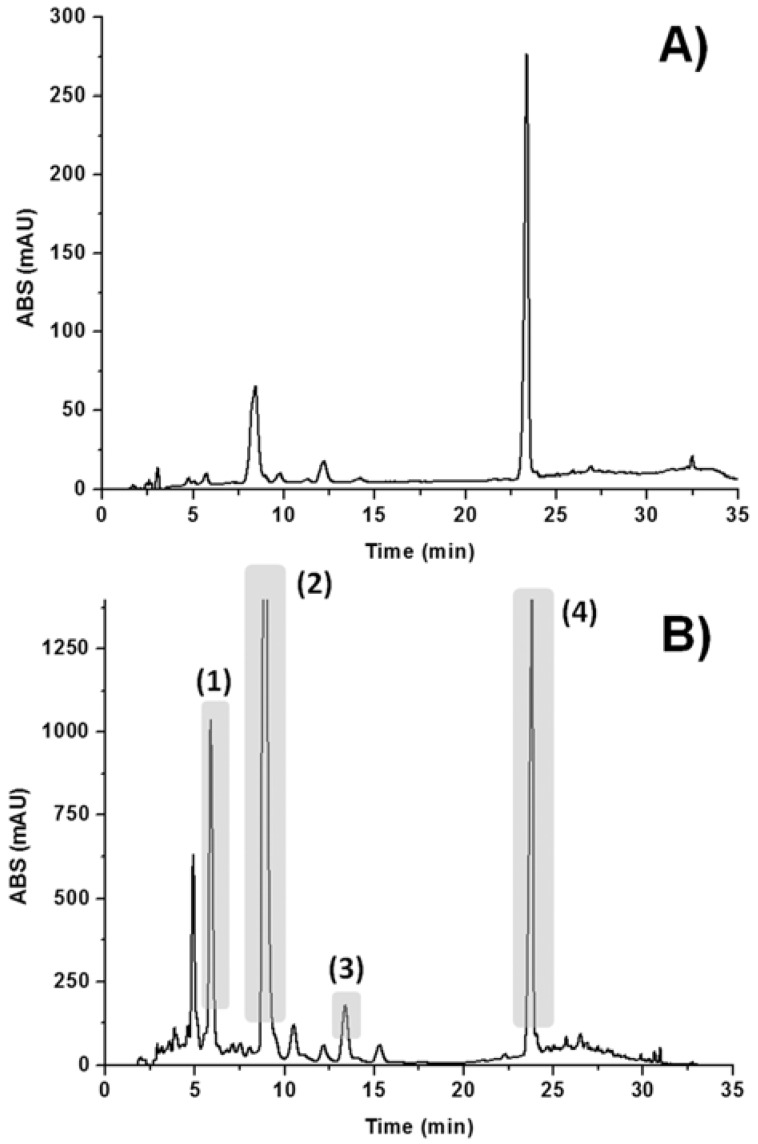
HPLC chromatograms of *Alternaria alternata* extract (UV detection at 312 nm): (**A**) concentration at 5 mg/mL; (**B**) concentration at 50 mg/mL.

A HPLC method has been developed to separate all interesting compounds contained in the extract obtained after fungal fermentation. Basically two peaks were detected with appropriate intensity when 5 mg/mL sample was injected (Figure 2A). However, when concentration was increased many additional peaks appeared in the chromatogram (Figure 2B). A concentration of 50 mg/mL was the highest value at which baseline separation was not significantly affected, so the extract at this concentration was injected (30 µL) six times into the LC-SPE-NMR system, which was done under automation, in order to trap as many peaks as possible on different SPE cartridges.

The ^1^H-NMR spectra of peaks **1** to **4** (Table 1) suggest metabolites related to alternariol. Measurements of the major components, *i.e.*, peaks **2** and **4**, resulted in very similar ^1^H-NMR data (Table 1). Four doublets at the aromatic region (δ 6.40 to 7.30) with *met*a-coupling constants suggest the presence of two tetrasubstituted aromatic rings. Intense singlets around δ 12.0 and 2.8 (integrating for 3H) indicate the presence of a chelated hydroxyl group and a methyl group linked to an aromatic ring, respectively. The only difference between the spectra of **2** and **4** is the presence of another 3H singlet at δ 3.92 in compound **4**, suggesting that a methoxyl group is attached to one of the aromatic rings. ^13^C-NMR spectra are also quite similar for both compounds **2** and **4**, except for the presence of a carbon signal at δ 56.7, which is assignable to the methoxyl group in compound **4**. HMBC correlations obtained for both compounds (Table 1) are similar as well and they corroborate to the presence of alternariol’s chemical skeleton. H-3' of **2** correlates with carbon atoms appearing at δ 110.2 and 153.9 (assigned as C-1' and C-2', respectively), and H-3' of **4** correlates with carbon atoms at δ 109.3 (C-1') and 153.4 (C-2'). The same can be observed for the connectivity between rings B and C of **2**, since H-6 (δ 7.28) correlates through three bonds with carbons at δ 110.2 and 99.6, assigned as C-1' and C-2, respectively. Such correlations of H-6 (δ 7.31) with C-1' (δ 109.3) and C-2 (δ 98.1) can be observed for compound **4** correspondingly. Based on these results and compared with published data [21,22], peaks **2** and **4** were identified as alternariol and alternariol monomethyl ether, respectively. High Resolution Electron Ionization Mass Spectrometry (HREIMS) gave exact masses which were consistent with the molecular formulae of compounds **2** and **4** (Table 2). Molecular ions [M^+^] at *m/z* 258.05197 (C_14_H_10_O_5_, cal.: 258.05281) were obtained for compound **2** and at *m/z* 272.06711 (C_15_H_12_O_5_, cal.: 272.0685) for compound **4**. Alternariol (**2**) and alternariol monomethyl ether (**4**) have been isolated from many different cultures of the genus Alternaria such as *A. alternata* [23], *A. arborescens*, *A. infectoria*, *A. tenuissima* [24], among others [25]. They exhibit mutagenic and cytotoxic effects to bacterial and mammalian cells [26], and they are suspected to be carcinogenic for human beings [10,11].

**Table 1:** NMR spectroscopic data (MeCN-*d_3_*, 500 MHz) for compounds **1**–**4**.

	Altenusin (1)					Alternariol (2)					
	Obtained			Reference (MeCN-*d*_3_) [26]		Obtained				Reference (CDCl_3_) [21]	
	δ_H_ (*J* in Hz)	δ_C_	HMBC	δ_H_ (*J* in Hz)	δ_C_	δ_H_ (*J* in Hz)	δ_C_		HMBC	δ_H_ (*J* in Hz)	δ_C_
**1**		147.2			148.3		139.0				138.1
**2**		105.3			106.5		99.6				97.4
**3**		165.7			163.0		164.9				164.1
**4**	6.47, d (2.6)	100.6	2, 3, 5, 6	6.25, d (2.2)	102.3	6.42, d (2.0)	101.1	2, 3, 6		6.38, d (1.9)	100.9
**5**		165.1			165.3		165.3				165.5
**6**	6.19, d (2.6)	111.4	1, 2, 4, 5	6.03, d (2.2)	112.1	7.28, d (2.0)	104.9	2, 4, 5, 1'		7.27, d (2.0)	104.4
**7**							165.1				164.7
**1'**		134.7			135.8		110.2				109.0
**2'**	6.52, s	116.4	1, 1', 3', 4', 6'	6.46, s	116.6		153.9				152.6
**3'**		142.5			143.3	6.68, d (2.4)	102.3	1', 2', 4', 5'		6.32, d (2.6)	101.6
**4'**		144.2			144.7		158.0				158.4
**5'**	6.63, s	117.1	1', 3', 4', 6'-CH_3_	6.55, s	117.2	6.73, d (2.4)	117.4	1', 3', 6'-CH_3_		6.71, d (2.6)	117.6
**6'**		127.6			127.3		139.2				138.3
**3-OH**	11.87, s					11.86, s		2, 3, 7			
**5-OCH_3_**	3.81, s	56.2	5	3.87, s	56.4						
**6'-CH_3_**	1.90, s	19.1	1', 2', 4' 5', 6'	1.90, s	19.3	2.78, s	25.4	1, 1', 5', 6'		2.77, s	25.3
	**3'-Hydroxyalternariol monomethyl ether (3)**					**Alternariol monomethyl ether (4)**					
	**Obtained**			**Reference (in DMF-*d*_7_) [26]**		**Obtained**				**Reference (in DMSO-*d*_6_) [22]**	
	**δ_H_ (*J* in Hz)**	**δ_C_**	**HMBC**	**δ_H_ (*J* in Hz)**	**δ_C_**	**δ_H_ (*J* in Hz)**	δ_C _*	**HMBC**		**δ_H_ (*J* in Hz)**	**δ_C_**
**1**		138.4			139.5		138.3				137.8
**2**		100.0			99.2		98.1				98.4
**3**		165.5			165.4		165.5				164.0
**4**	6.52, d (1.9)	100.3	2, 3, 6	6.63, d (1.8)	99.7	6.57, d (2.0)	101.5	2, 3, 5, 6		6.59, d (2.5)	101.6
**5**		167.5			167.3		166.5				164.6
**6**	7.31, d (1.9)	104.8	2, 4, 5, 1'	7.30, d (1.8)	104.2	7.31, d (2.0)	103.3	2, 4, 5, 1'		7.20, d (2.5)	103.5
**7**		n.d.			165.8		168.3				166.1
**1'**		111.4			110.1		109.3				108.3
**2'**		142.2			142.3		153.4				152.6
**3'**		131.2			132.4	6.74, d (2.4)	101.5	1', 2', 4', 5'		6.63, d (2.0)	99.1
**4'**		146.4			148.2		158.9				158.5
**5'**	6.76, s	117.5	1', 2', 3', 4', 6', 6'-CH_3_	6.82, s	117.7	6.68, d (2.4)	117.5	1', 3', 4', 6'-CH_3_		6.71, d (2.0)	117.5
**6'**		128.7			123.3		138.8				138.4
**3-OH**	11.91, s					11.89, s					
**5-OCH_3_**	3.92, s	56.6	5	3.99, s	56.3	3.92, s	56.7	5		3.90, s	55.8
**6'-CH_3_**	2.70, s	25.1	1, 6, 1', 2', 5', 6'	2.74, s	24.9	2.78, s	25.7	1, 1', 2', 5', 6'		2.72, s	24.6

* Obtained from HSQC and HMBC experiments obtained in MeOD-*d_4_*.

By increasing the sample concentration injected onto the column it was possible to identify two more peaks, labeled herein as **1** and **3** (Figure 2B). Compound **1** shows a similar ^1^H-NMR spectrum if compared with compounds **2** and **4**, although aromatic signals are shifted to a more shielded region (Table 1). However, two of the doublets observed in the ^1^H-NMR spectra of **2** and **4** collapsed into two singlets in the ^1^H-NMR spectrum of **1**. These hydrogen atoms (H-2' and H-5') exhibit HMBC correlation with carbon atoms C-3' and C-4', suggesting a change in the substitution pattern of one of aromatic rings. Furthermore, a shift of both ^1^H and ^13^C signals attributed to the methyl group at ring A from δ 2.78 and 25.4, respectively, in **2** to a more shielded region at δ 1.90 and 19.1 in compound **1** was observed. Such a shift is consistent with an opening of the lactone ring [27]. Moreover, a HMBC correlation between H-2' (δ 6.52) and C-6' (δ 127.6) in compound **1** is also evidence of such opening (Table 1). Thus, based on this data and on comparison with literature [27], compound **1** was identified as altenusin (Figure 1), a metabolite produced by *Alternaria* and other microorganisms [28]. Once again HREIMS supported the structure proposition since a molecular ion at *m/z* 290.07970 (C_15_H_14_O_6_, cal.: 290.07904) was observed (Table 2).

**Table 2:** HREIMS data for compounds **1**–**4**.

Compound	Detected ion (*m/z*)	Calculated mass (Da)	Accuracy (ppm)	Deviation (mDa)
Altenusin (1) (C_15_H_14_O_6_)	290.07970	290.07904	−2.3	−0.7
Alternariol (2) (C_14_H_10_O_5_)	258.05198	258.05281	3.3	0.8
3'-Hydroxyalternariol monomethyl ether (3) (C_15_H_12_O_6_)	288.06381	288.06339	−1.5	−0.4
Alternariol monomethyl ether (4) (C_15_H_12_O_5_)	272.06711	272.06847	5.0	1.4

Altenusin (**1**) is reported as a less toxic compound than its analogs alternariol (**2**) and alternariol monomethyl ether (**4**). However, Nakanishi *et al.* [28] verified that it could act as a possible inhibitor of myosyl light chain kinase, an enzyme that triggers contraction of smooth muscles. In addition, Aly *et al.* [27] reported that altenusin exhibits a considerable cytotoxic activity against mouse lymphoma cells, with a value for half maximal effective concentration (EC_50_) of 23.4 nM.

Compound **3** exhibited a very similar ^1^H-NMR data when compared to alternariol monomethyl ether (**4**) (Table 1). The main difference is the absence of two doublets at δ 6.78 and 6.72. Instead, it displayed a singlet at δ 6.80, which suggests the presence of a new substituent attached to the ring A.

According to the HREIMS results, which gave a molecular ion at *m/z* 288.06339 (C_15_H_12_O_6_, cal.: 288.06339), the new substituent could be a hydroxyl group (Table 2). HMBC correlations also matched the proposed structure of compound **3** (Table 2). The hydrogen signal at δ 6.76 (H-5') in the spectrum of compound **3** strongly correlates with the carbon signals at δ 25.1 (6'-CH_3_), δ 111.4 (C-1') and δ 131.2 (C-3'). These correlations indicate that the additional hydroxyl group is attached at position 3' in compound **3**, which was identified as 3'-hydroxyalternariol monomethyl ether. This compound was already isolated from *Alternaria* culture [26].

**Figure 3:**
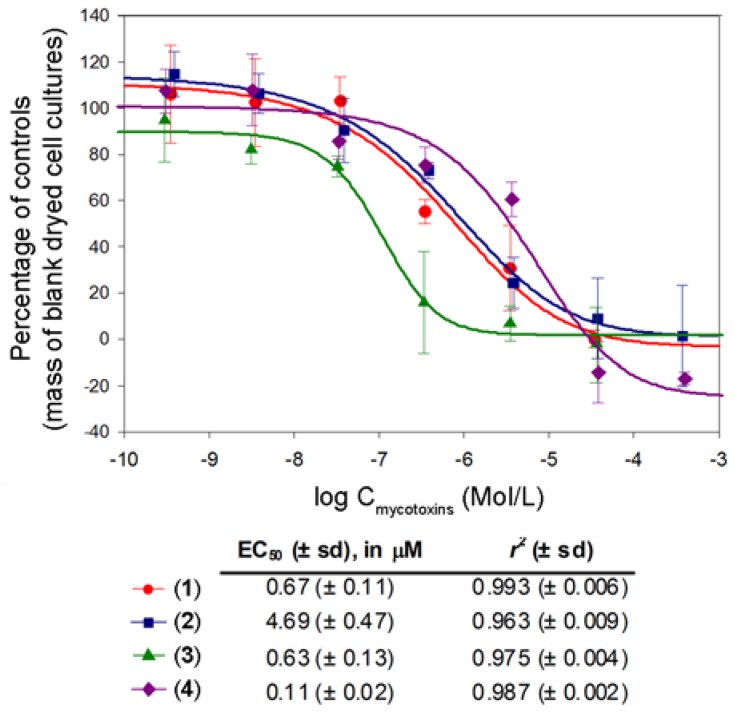
Cytotoxicity assay for compounds **1**–**4** using soybean cell suspension cultures. EC_50_ values were calculated using the four parameters logistic model equation. The obtained values ranged from 110 nM to 4.69 µM, which highlights the cytotoxic effect of those compounds.

In order to verify whether such compounds exhibit toxic effects on plant cells, soybean cell suspension cultures were used to develop a biological assay. Cell cultures have been using for predicting mycotoxins effects for over two decades [29]. Results obtained herein are presented in Figure 3. As it can be seen, all compounds **1**–**4** exhibited cytotoxic properties with half maximal effective concentrations (EC_50_) at nM and µM levels. In addition, the four parameter logistic model, which was the statistical model employed to determine cytotoxic properties of interesting compounds, has matched with obtained results with r^2^ greater than 0.96.

Altenusin (**1**) and alternariol (**2**) showed similar EC_50 _ values, suggesting that the presence of a lactone ring is not crucial for cytotoxic effects. However, the presence of this ring on such kinds of structures is apparently closely related to cytotoxic properties since similar structures such as deoxytalaroflavone and 1-deoxyrubralactone, in which the ring A is contracted to a 5-membered ring, exhibit no cytotoxic effects [30,31]. Phenolic hydroxyl groups apparently play a key role in toxicity for soybean cell culture as well. If alternariol (**2**) is converted to alternariol monomethyl ether (**4**), the effect is apparently decreased sevenfold. Instead, if a hydroxyl group is introduced on ring A of **4** to give 3'-hydroxyalternariol monomethyl ether (**3**), cytotoxic effects are increased nearly 43-fold.

Few reports concerning cytotoxic effects of alternariols can be found in the literature. Aly *et al.* [26] reported strong effects of altenusin (**1**), alternariol (**2**), and alternariol monomethyl ether (**4**) towards mouse lymphoma cells. Alternariols have exhibited cytotoxic effects against both animal [26] and human mammalian cells [32]. However, this is apparently the first report concerning toxicity to vegetal cells.

## 3. Experimental

### 3.1. General Experimental Procedures

All LC-SPE-NMR analyses were carried out in a system composed of an Agilent Series 1100 liquid chromatograph (Agilent, Waldbronn, Germany) coupled to a Prospekt II SPE unit (SparkHolland, Emmen, The Netherlands). This system was coupled to an Avance 500 Ultrashield NMR equipment (11.7 T, Bruker, Rheinstetten, Germany) equipped with a TCI cryoprobe (5 mm) and a Cryofit insert for detection (flow cell volume 30 μL). A make-up pump (Knauer, Berlin, Germany) was used to add water (2.5 mL min^−1^) to the eluent after HPLC in order to reduce the eluotropic capacity. For HREIMS spectra, a Micromass MasSpec with three-sector double focusing MS (Waters, Milford, MA, USA) was used.

### 3.2. Fungal Cultivation and Preparation of Extracts

The fungus *Alternaria alternata* was isolated as previously reported [5] and cultivated in 15 flasks containing 70 g of wheat (Yoki, São Bernardo do Campo, Brazil) and 50% (v/w) of distillated water. The cultures were incubated at 25 °C for 25 days. After fungal growth, the contents of each flask were extracted twice with 200 mL of ethyl acetate (JT Baker, Ecatepec, Mexico) and twice with 200 mL of methanol (JT Baker). Extracts were then pooled and evaporated using a R-210 rotary evaporator (Büchi, Flawil, Switzerland).

### 3.3. Separation and Trapping of Alternaria Metabolites

Separation of the compounds was achieved employing gradient elution using acetonitrile (Merck, Darmstadt, Germany) with 0.1% of trifluoroacetic acid (TFA) (Merck) (solvent B) and ultrapure water (RiOs method, Millipore) with 0.1% of TFA (solvent A). For separating interesting compounds a Lichrosorb RP-18 column (250 × 4.6 mm, 5 µm, Merck) was used and 20 µL of extract were injected in each analysis. The flow of mobile phase was set at 1 mL/min and UV detection was done at both 256 and 312 nm. The method employed a gradient elution starting with 37% of solvent B from 0 to 17 min. Then, the mobile phase linearly changed to 55% in 5 min. From 22 to 25 min, the mobile phase varied from 55 to 95%. The column was cleaned for 10 min and adjusted to the initial composition of mobile phase in 2 min. Conditioning time was set at 10 min. The stationary phase of SPE cartridges was a Hysphere resin GP 10–12 μm (SparkHolland, Emmen, The Netherlands), which is a polydivinyl-benzene resin that can be used for the retention of a wide variety of organic compounds. Samples were injected from 6 to 8 times and peaks detected at identical retention times trapped on the same SPE cartride in order to retain as much compound as necessary.

### 3.4. NMR and MS analyses

All trapped peaks were eluted from the SPE cartridges using 240 μL of MeCN-*d_3_* (Deutero, Kastellaun, Germany). ^1^H-NMR and 2D spectra (HSQC, HMBC) were acquired using standard Bruker pulse sequences (Topspin 2.0, Bruker, Rheinstetten, Germany). The number of scans was determined according to the compound concentration and varied from 32 to 5,120. Spectra processing was done using zero filling (SI ≥ TD) and exponential multiplication (lb) of 0.3. Correlations obtained in HMBC experiments are from hydrogen(s) stated to the indicated carbons on the Table 1. NMR spectra were referenced to the signals of the solvent, MeCN-*d_3_* (^1^H-NMR: δ 1.93; ^13^C-NMR: δ 1.39). Carbon chemical shift values were extracted from HMBC or HSQC experiments. For alternariol monomethyl ether (**4**) ^13^C-NMR values were extracted from HMBC and HSQC experiments recorded in MeOD-*d_4_* (^1^H-NMR: δ 3.31; ^13^C-NMR: δ 49.1). After acquiring NMR experiments, compounds were collected using a fraction collector (C-LAB Chemievermittlung, Birkenfeld, Germany) and dried under N_2_ flow. Then, samples were weighted and submitted for HREIMS analysis and biological tests.

### 3.5. Cytotoxic (Phytotoxic) Assays

Soybean (*Glycine max* L. cv. Harosoy 63) cell suspension cultures were grown according to Fliegmann *et al.* [27]. After 5 days of growth 1 mL of suspension culture was carefully transferred to each well of 24 well plates (Costar 3526, Corning Inc., New York, NY, USA). Different solutions of interesting compounds in 96% ethanol (Merck) were added to each well so that final concentrations were at 0.1, 1.0, 10, 100, 1000, and 10,000 ng/mL. Every well was left with the same final volume and with the same amount of solvent (1.8%). All points were registered fivefold for each concentration. In addition, wells containing cell cultures plus solvent were maintained as control (also fivefold). All 24 well plates were kept under agitation and in the dark for 48 h. Soybean cell culture suspensions were then carefully transferred to 1.5 mL Eppendorf tubes (Eppendorf, Hamburg, Germany), freeze-dried for 24 h (Heto Maxi-Dry Lyo, Jouan Nordic A/S, Allerod, Denmark), and finally the dried mass of each well was determined. Such experiments were repeated threefold and compared with controls (cell cultures solution and cell cultures plus solvent). EC_50_ value calculations were done using the four parameter logistic model. Coefficients of variation as well as r^2^ values were achieved using SigmaPlot 2001 (SPSS, Chicago, IL, USA).

## 4. Conclusions

The present investigation demonstrated the usefulness of the LC-SPE-NMR as a fast approach for metabolites identification in crude extracts and provides comparative data on the cytotoxic effects of four Alternaria toxins on soybean cells. As plant cell suspension cultures can be a model for investigating toxicity for plants in general [33], those results indicate that compounds **1**–**4** might have a considerable phytotoxic effect. These outcomes suggest that alternariols could play a key role on the plant-pathogen interaction, probably as non-specific phytotoxins.

## Supporting Information

NMR (1D and 2D) and HREIMS spectra of detected compounds are available as supplementary materials, which can be accessed at: http://www.mdpi.com/1420-3049/18/3/2528/s1.
