# Supplementary material for: Identification of Alternaria alternata Mycotoxins by LC-SPE-NMR and Their Cytotoxic Effects to Soybean (Glycine max) Cell Suspension Culture
Source: Molecules. 2013 Feb 26;18(3):2528–38. doi: 10.3390/molecules18032528 (PMC6270395; doi:10.3390/molecules18032528)

Supplementary Materials

Figure S1. ^{1}H-NMR spectrum of altenusin (1) (CD_3CN, 500 MHz).

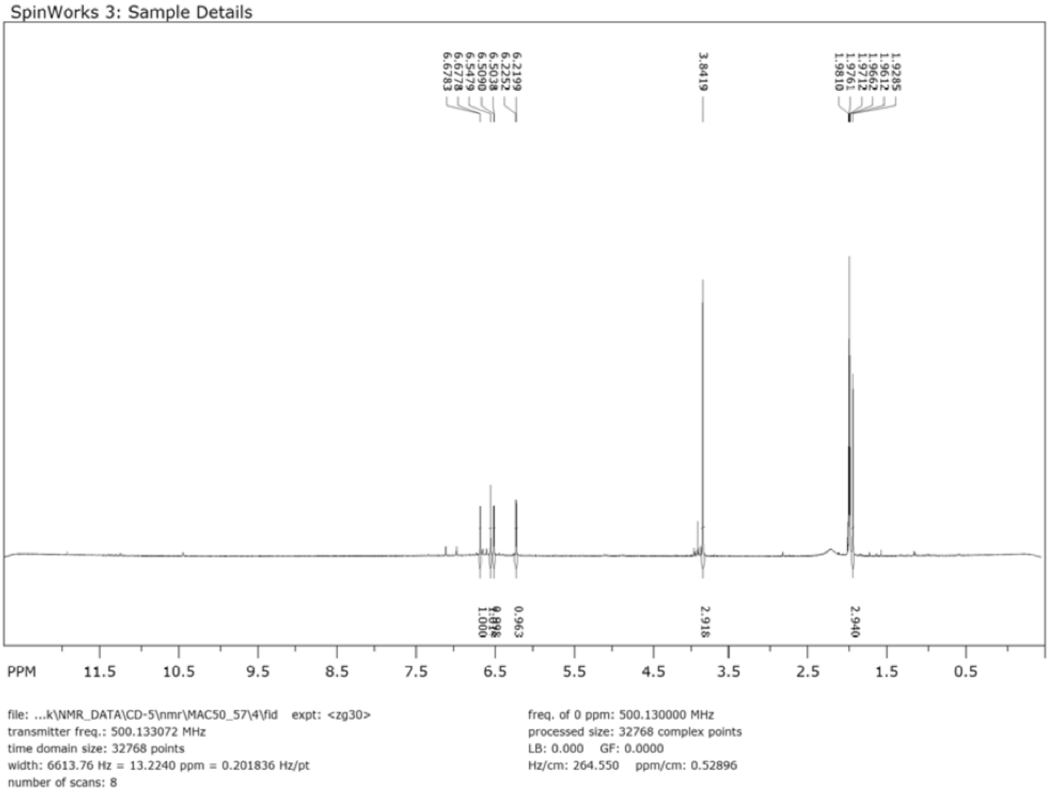

Figure S2. ^{1}H-NMR spectrum of altenusin (1)—from 6.20 to 6.70 ppm (CD_3CN, 500 MHz).

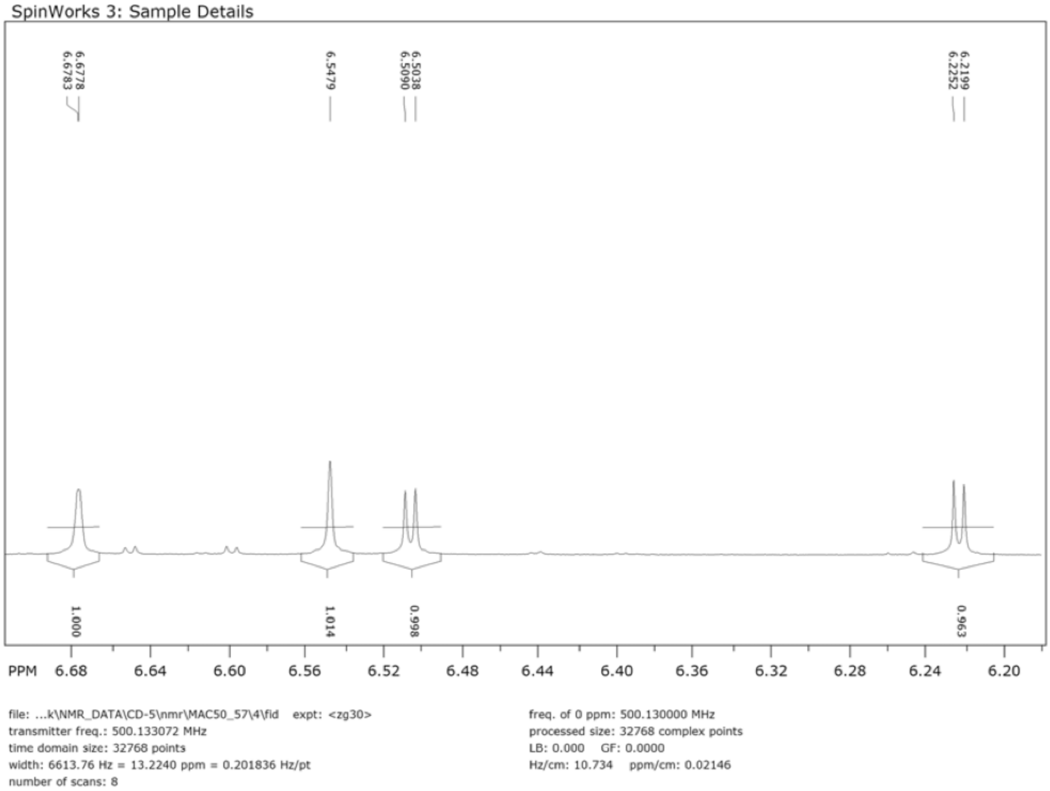

Figure S3. ^{1}H-NMR spectrum of altenusin (1)—from 1.80 to 4.10 ppm (CD_3CN, 500 MHz).

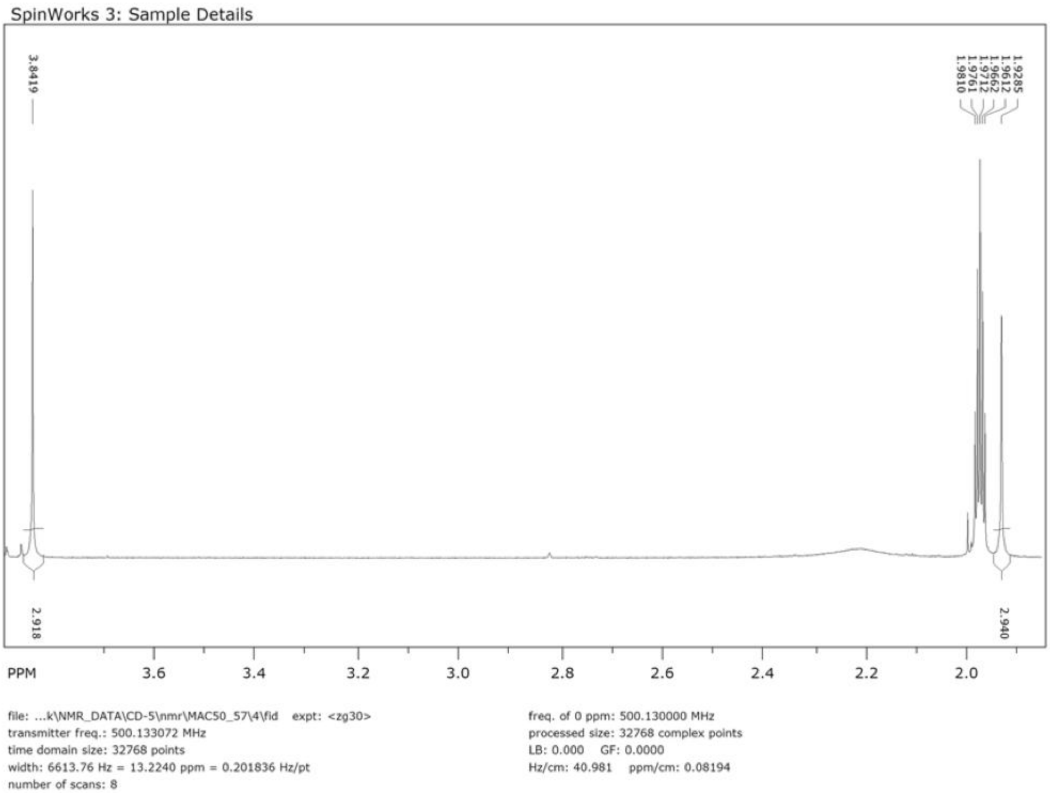

Figure S4. HSQC spectrum of altenusin (1) (CD_3CN, 500 MHz).

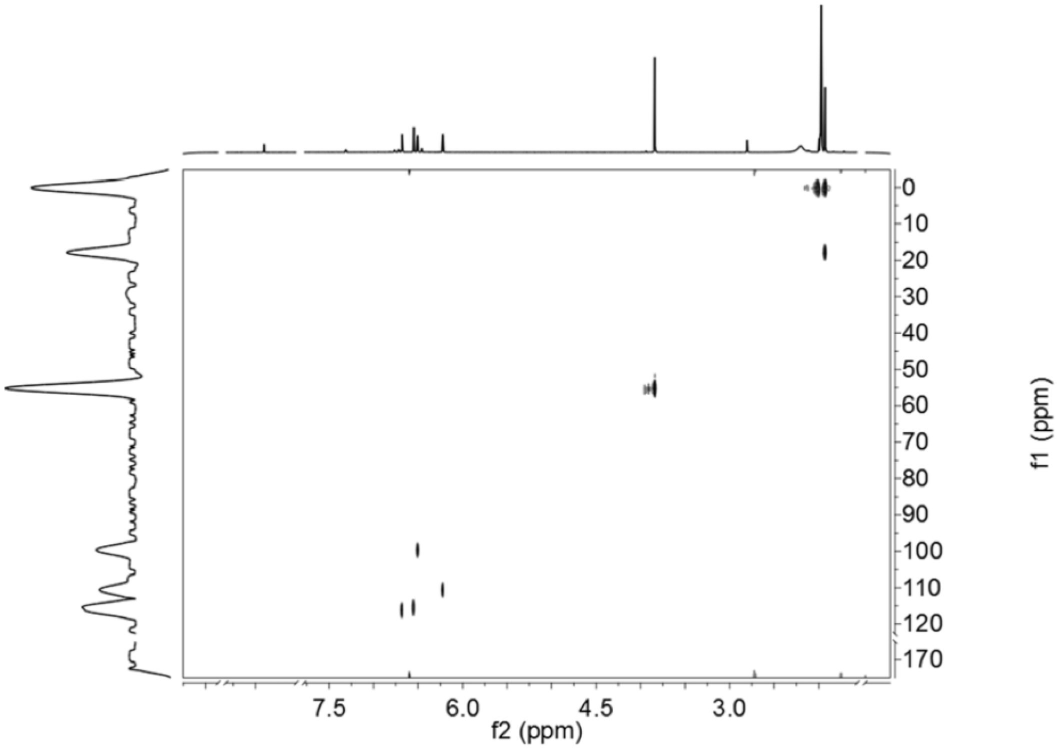

Figure S5. HMBC spectrum of altenusin (1) (CD_3CN, 500 MHz).

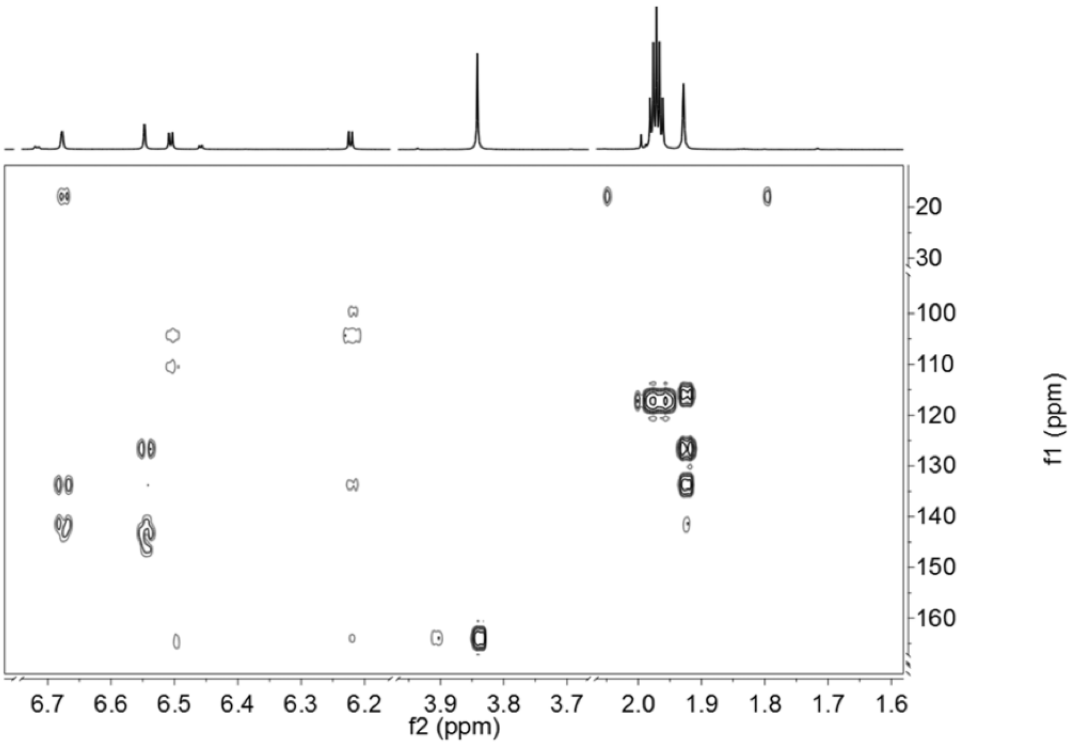

Figure S6. ^{1}H-NMR spectrum of alternariol (2) (CD_3CN, 500 MHz).

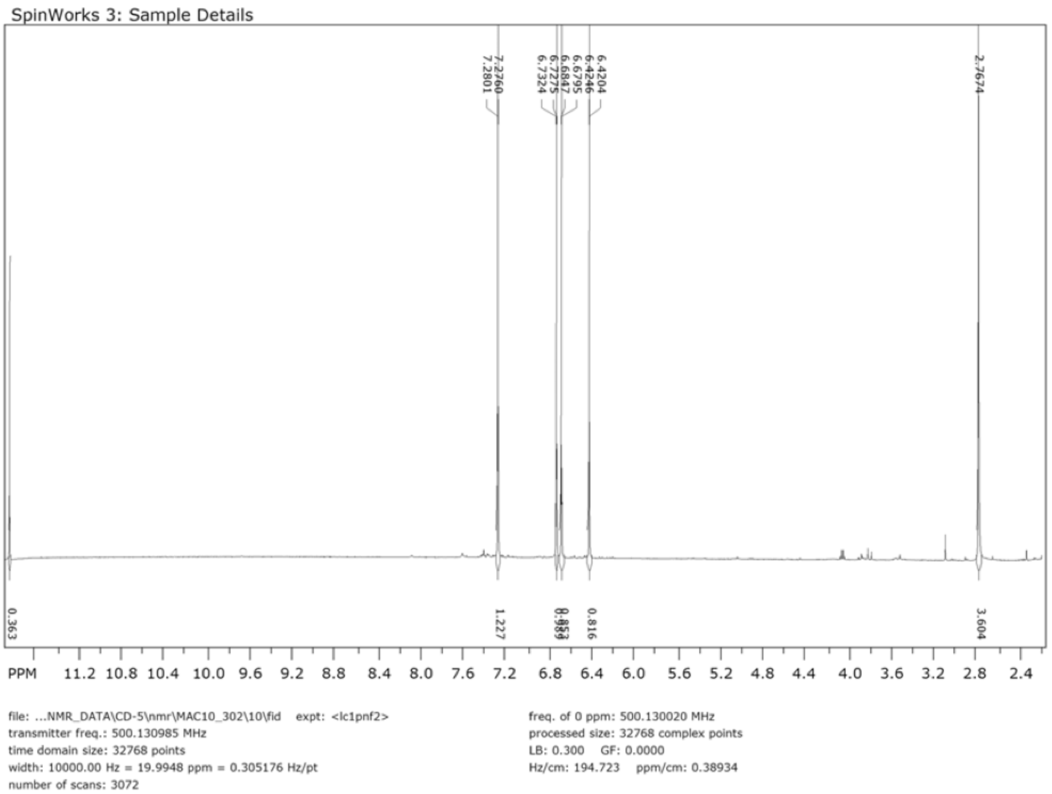

Figure S7. ^{1}H-NMR spectrum of alternariol (2)—from 6.35 to 7.32 ppm (CD_3CN, 500 MHz).

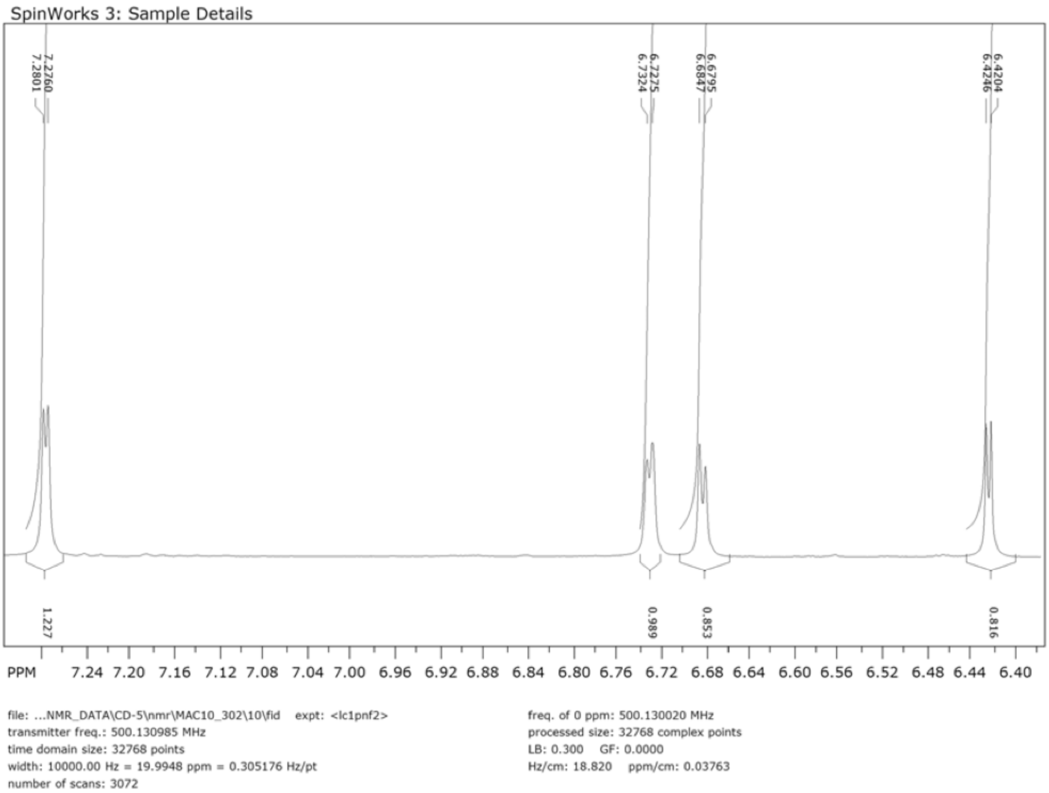

Figure S8. HMBC spectrum of alternariol (2) (CD_3CN, 500 MHz).

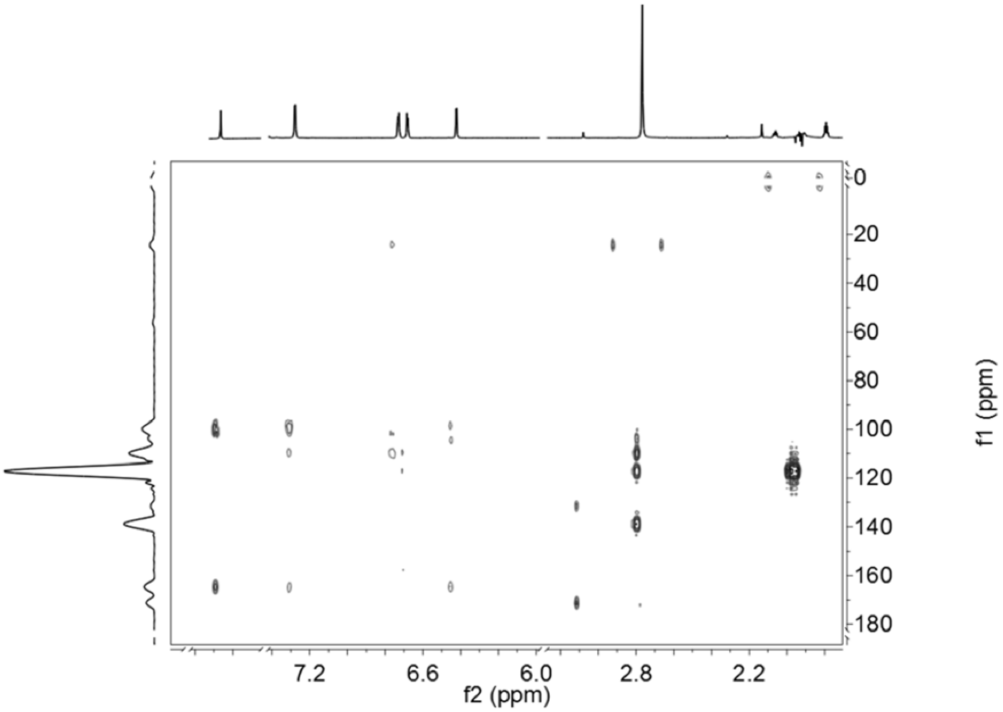

Figure S9. ^{1}H-NMR spectrum of 3'-hydroxyalternariol monomethyl ether (3) (CD_3CN, 500 MHz).

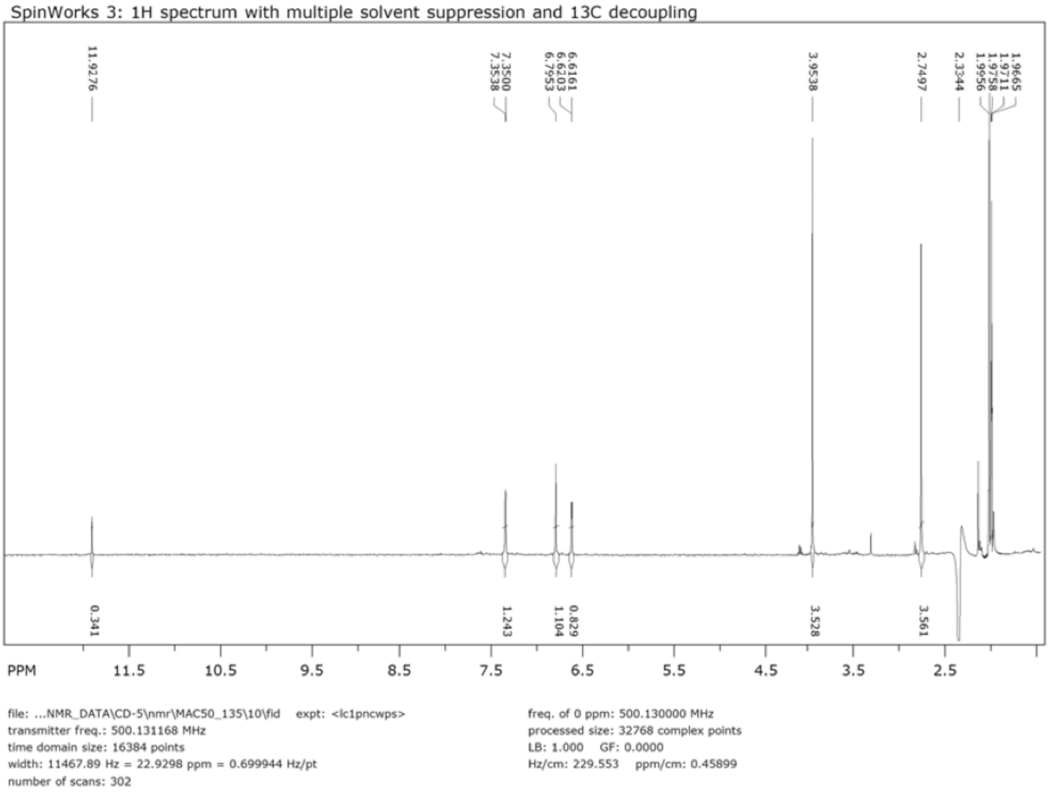

Figure S10. ^{1}H-NMR spectrum of 3'-hydroxyalternariol monomethyl ether (3)—from 6.55 to 7.50 ppm (CD_3CN, 500 MHz).

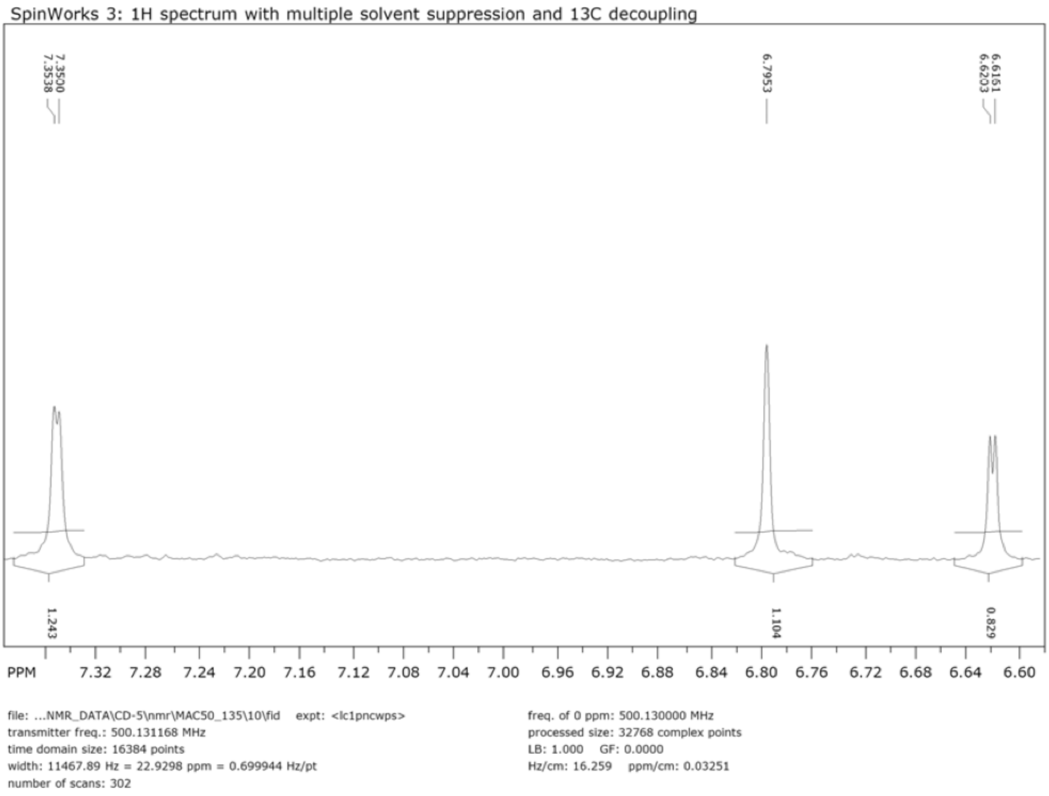

Figure S11. HSQC spectrum of 3'-hydroxyalternariol monomethyl ether (3) (CD_3CN, 500 MHz).

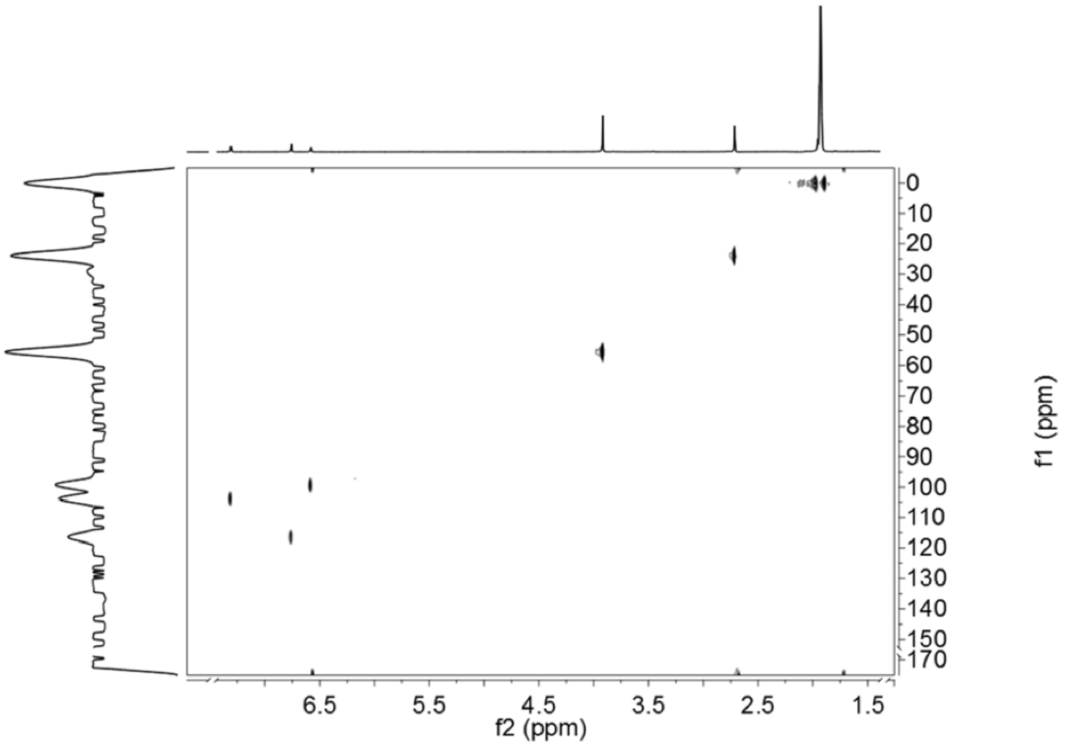

Figure S12. HMBC spectrum of 3'-hydroxyalternariol monomethyl ether (3) (CD_3CN, 500 MHz).

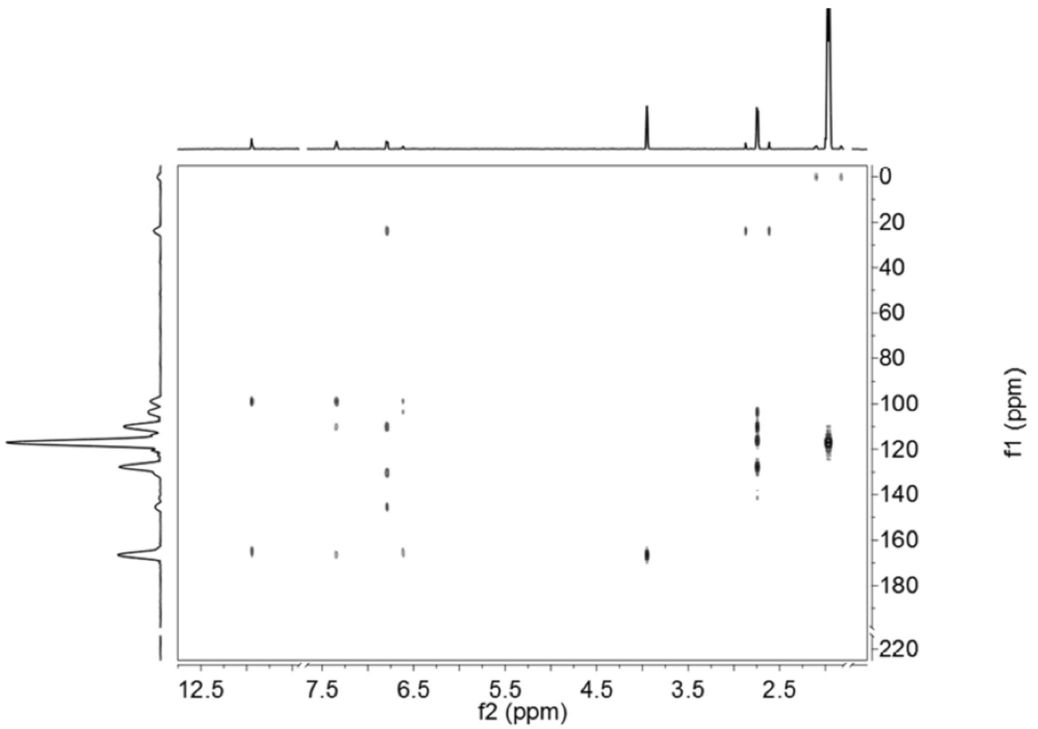

Figure S13. ^{1}H-NMR spectrum of alternariol monomethyl ether (4) (CD_3CN, 500 MHz).

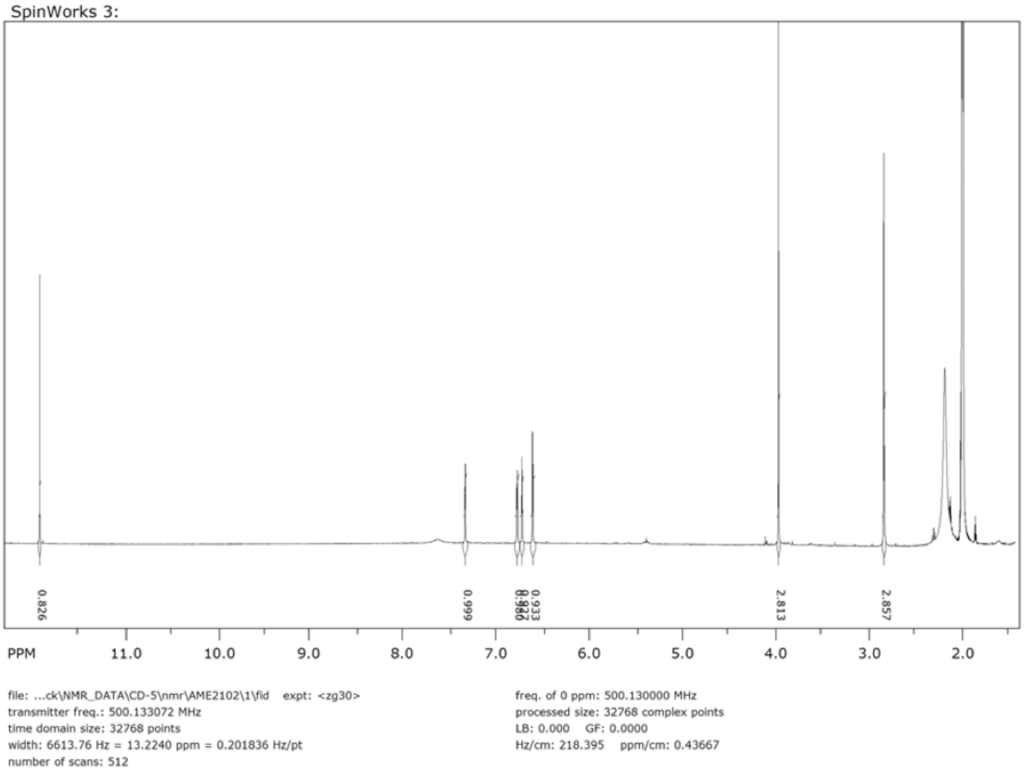

Figure S14. ^{1}H-NMR spectrum of alternariol monomethyl ether (4)—from 6.50 to 7.50 ppm (CD_3CN, 500 MHz).

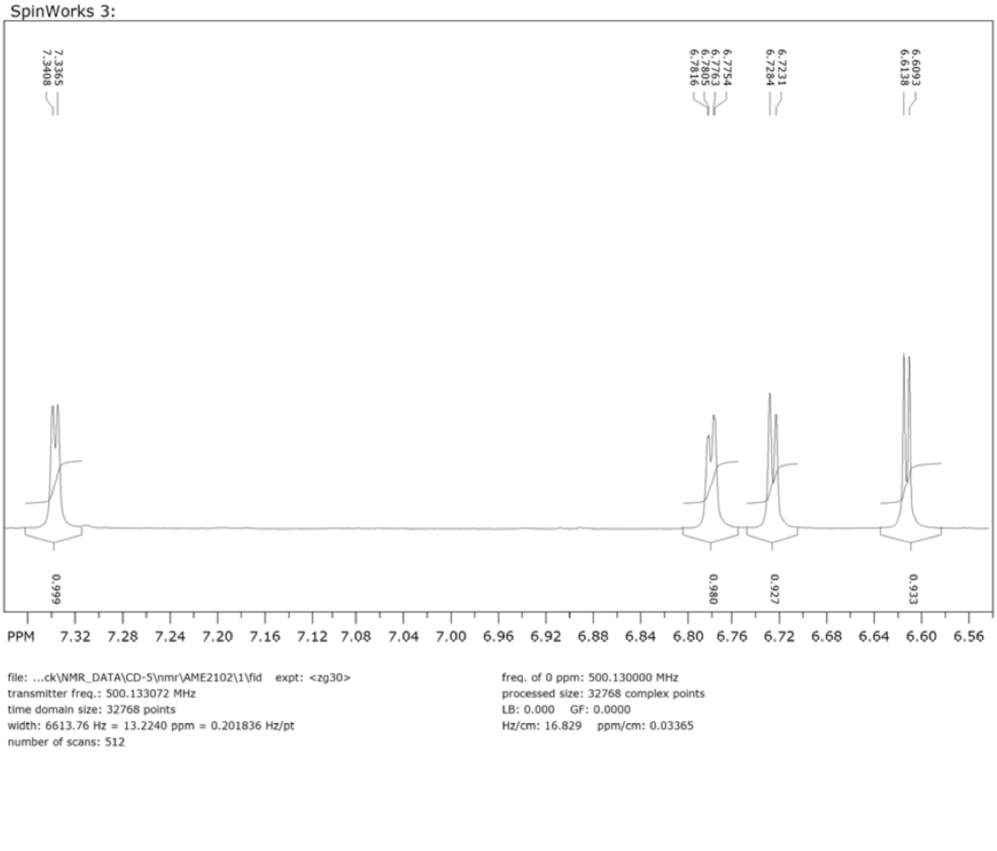

Figure S15. ^{13}C-NMR spectrum of alternariol monomethyl ether (4) (CD_3CN, 125 MHz).

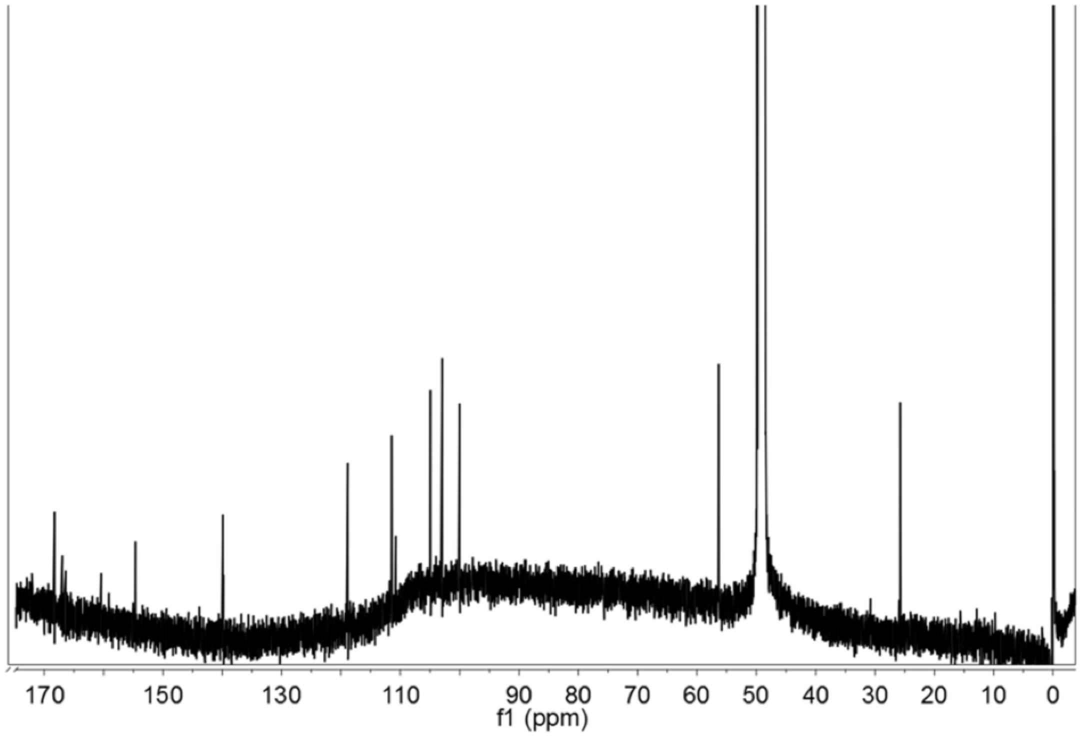

Figure S16. HMBC spectrum of alternariol monomethyl ether (4) (CD_3CN, 500 MHz).

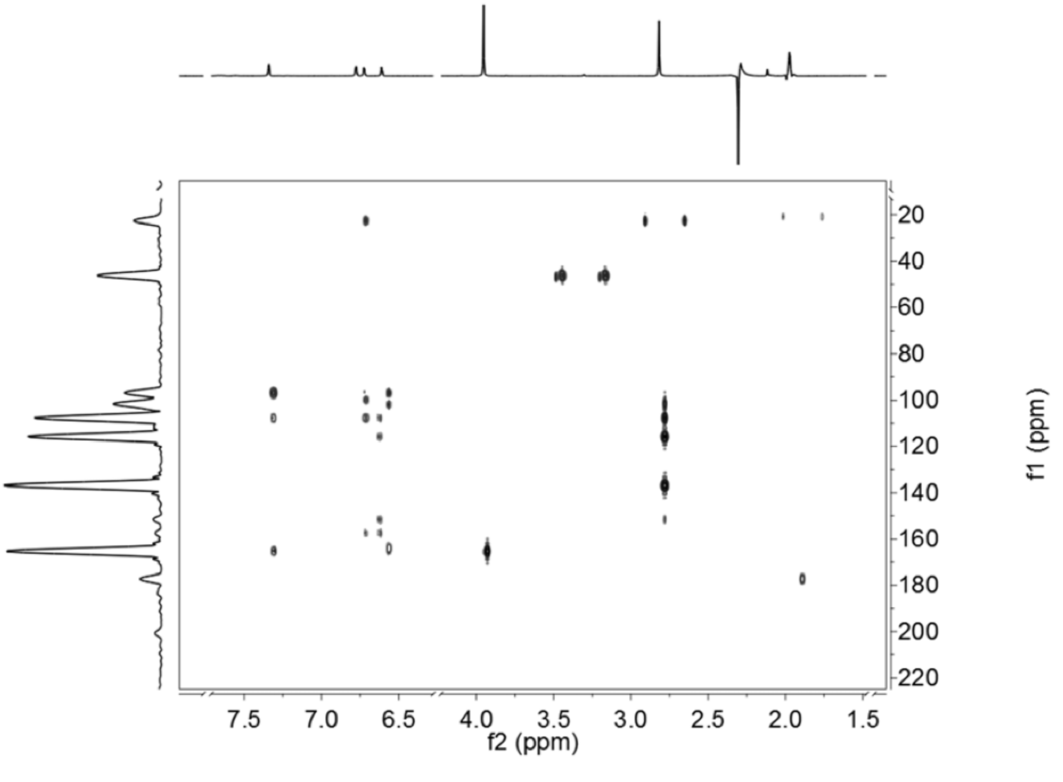

Figure S17. High Resolution Mass Spectrum (EI+) of alternusin (1).

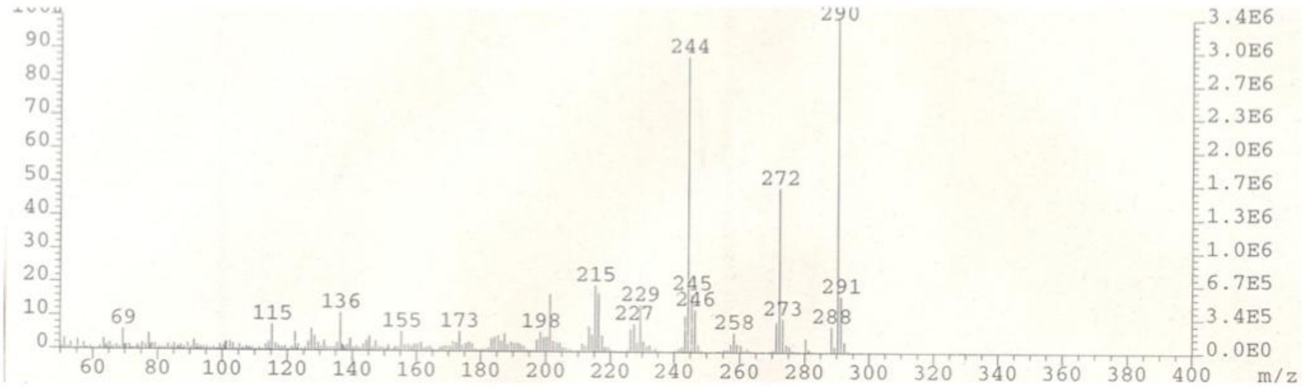

Figure S18. High Resolution Mass Spectrum (EI+) of alternariol (2).

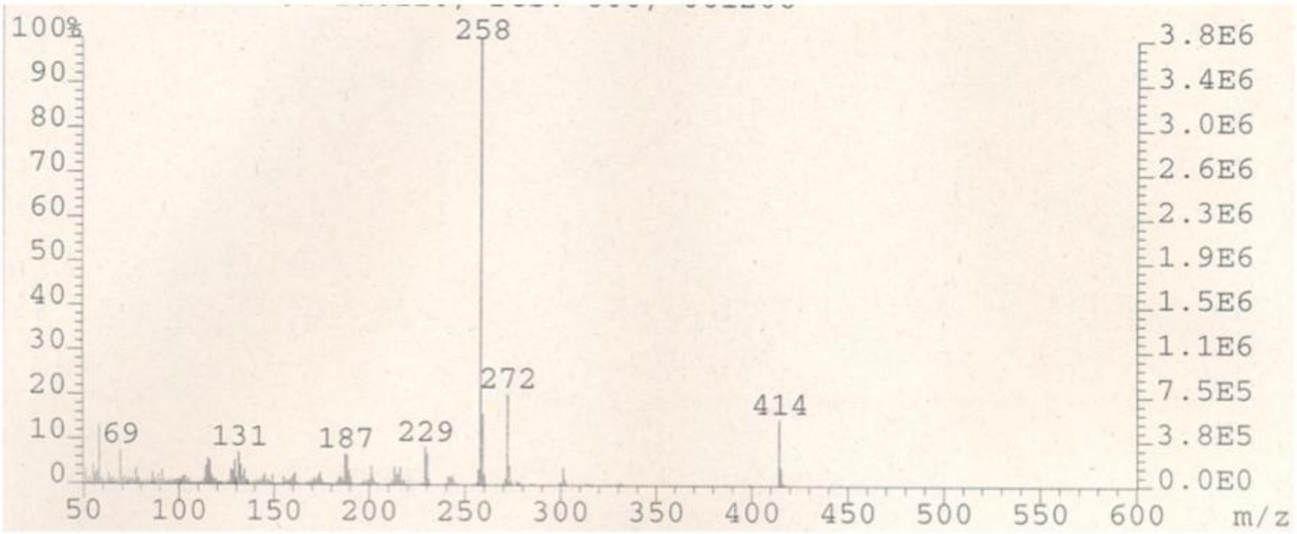

Figure S19. High Resolution Mass Spectrum (EI+) of 3'-hydroxyalternariol monomethyl ether (3).

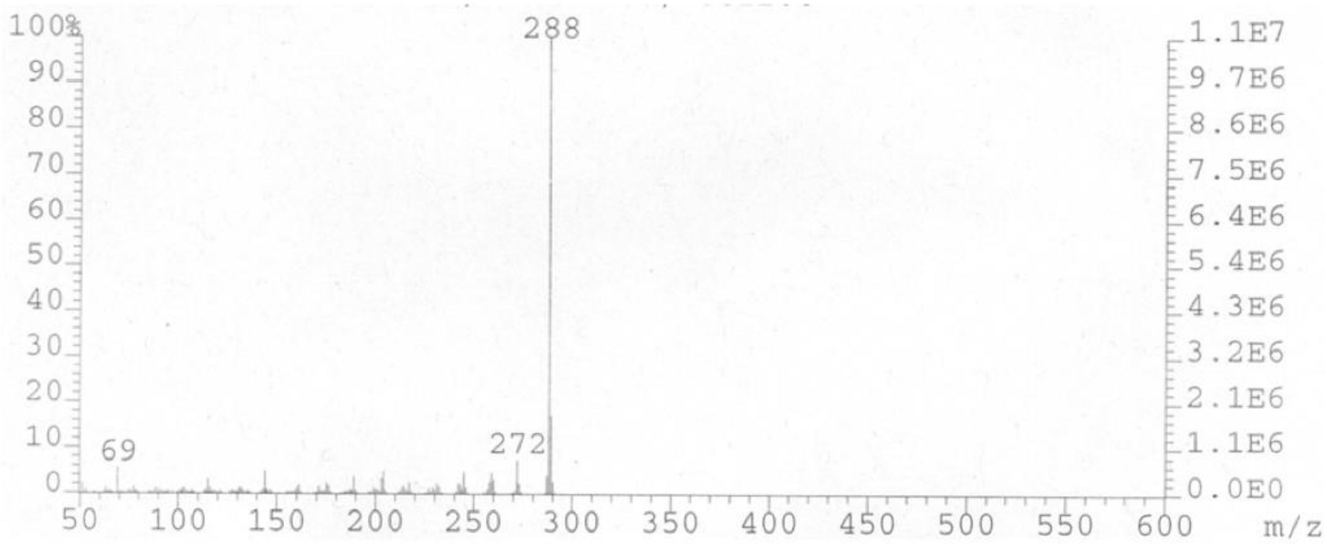

Figure S20. High Resolution Mass Spectrum (EI+) of alternariol monomethyl ether (4).

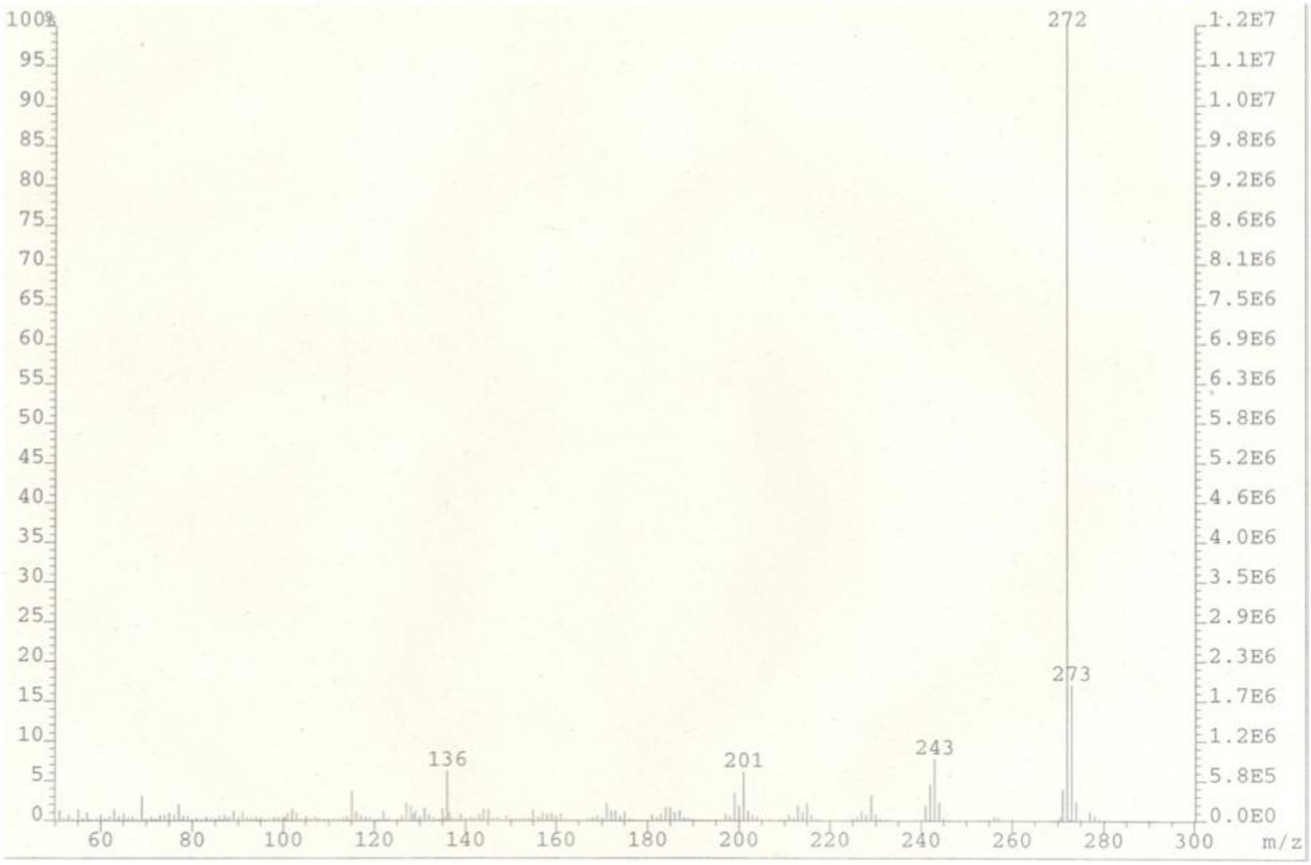

Supplement: Supplementary file 1 [file molecules-18-02528-s001.pdf]
